# Supplementary material for: In Vivo Modelling of Hepatitis B Virus Subgenotype A1 Replication Using Adeno-Associated Viral Vectors
Source: Viruses. 2021 Nov 9;13(11):2247. doi: 10.3390/v13112247 (PMC8618177; doi:10.3390/v13112247)
Supplement: Supplementary file 1 [file viruses-13-02247-s001.zip › viruses-1428681-supplementary.pdf]

viruses

Supplementary material

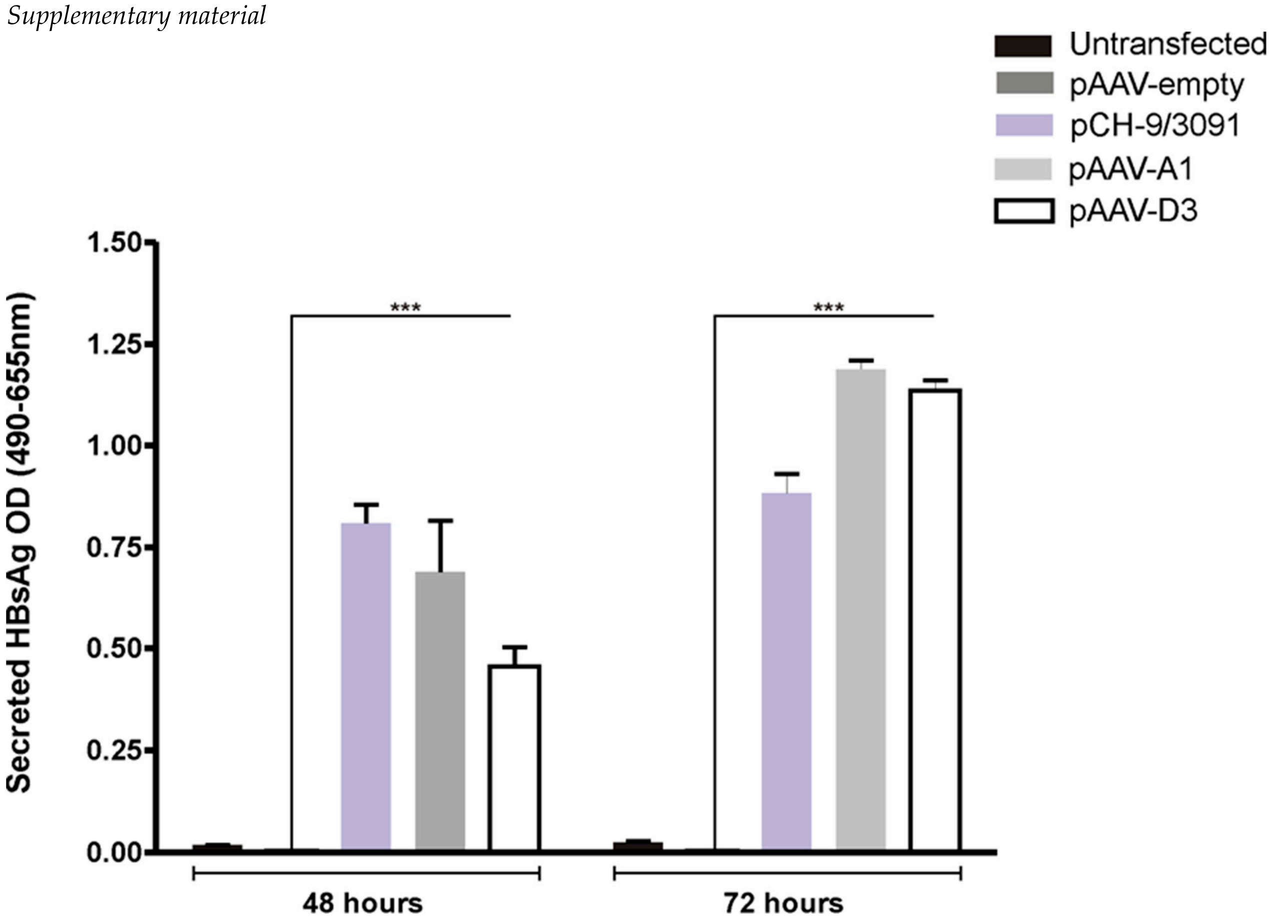

Figure S1. HBsAg expression in liver derived cells transfected with pAAV plasmids. Data were expressed as mean and SEM of four replicates. The statistically significant difference to AAV empty was determined using student two tailed paired t-test. ***$P \leq 0.005$ was considered as significantly different.

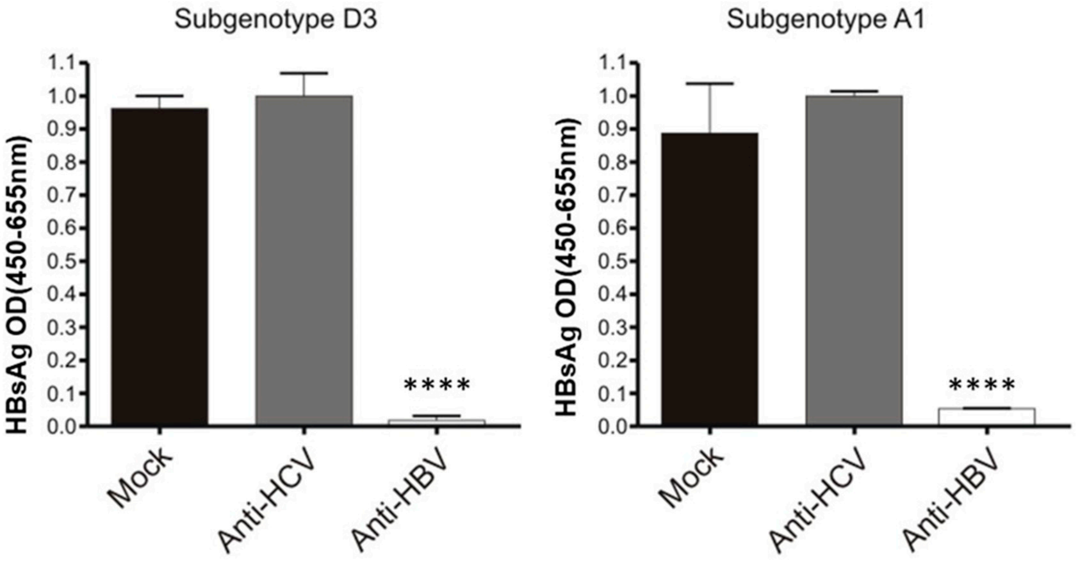

Figure S2. Inhibition of HBV gene expression from AAV2-HBV vectors in vitro. Huh 7 cells were co-infected with AAV2-HBV and pri-miR expressing vector or with AAV2-HBV only (mock) at an MOI of 1×10^4 each. The quantification was normalised to data from anti-HCV co-infected cells. ELISA was performed at 48 h post co-infection with AAV vectors. The means and SEM were derived from triplicate values. Statistically significant differences between samples were calculated using Student's two tailed paired t-test. ****$P \leq 0.0001$.

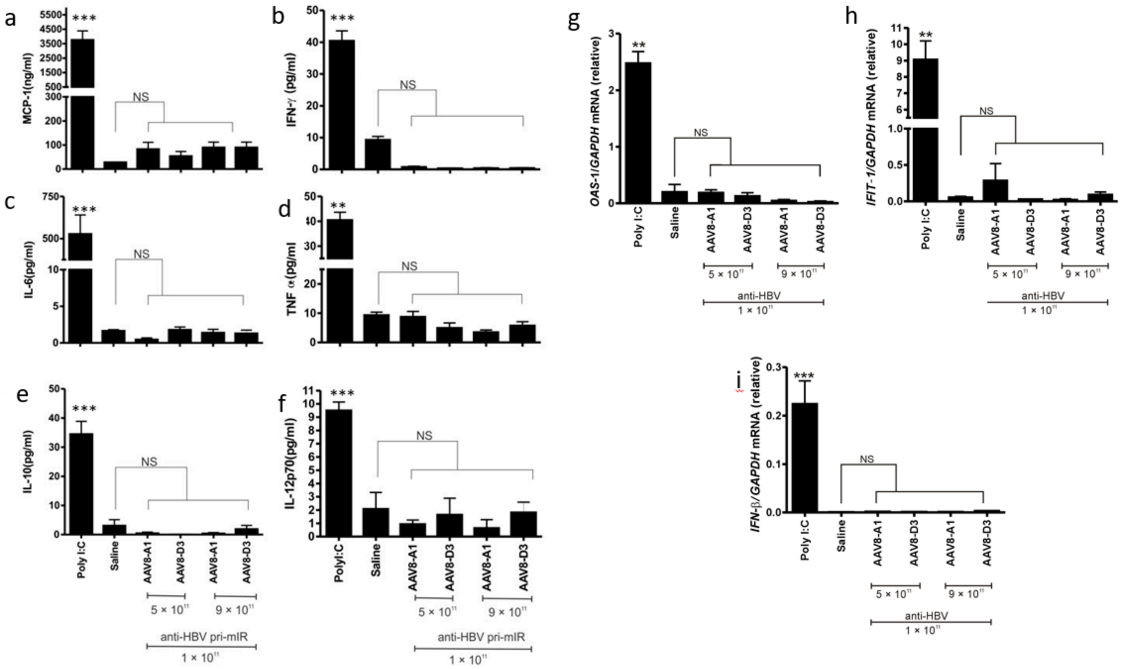

Figure S3. Stimulation of the IFN response in NMRI mice following intravenous co-administration of the AAV-HBV and pri-miR-expressing AAV. Levels of a, chemoattractant protein 1 (MCP-1), b, interferon gamma (IFN- γ), c, interleukin 6 (IL-6), d, tumour necrosis factor alpha (TNF-α), e, interleukin 10 (IL-10) and f, interleukin 12p70 (IL-12p70) cytokines 6 h post co-infection of AAV vectors or injection with Poly (I:C) or saline. g-i, OAS-1, IFN-β and IFIT-1 gene expression relative to GAPDH gene expression in mouse livers collected 6 h co-infection of AAV vectors or injection with Poly (I:C) or saline. Values represent the means and SEM calculated from injection of four mice per group. Statistically significant differences between samples were calculated using Student's two tailed paired t-test. ***P* ≤0.01; ****P* ≤0.001 was considered statistically significant NS: non-significant.

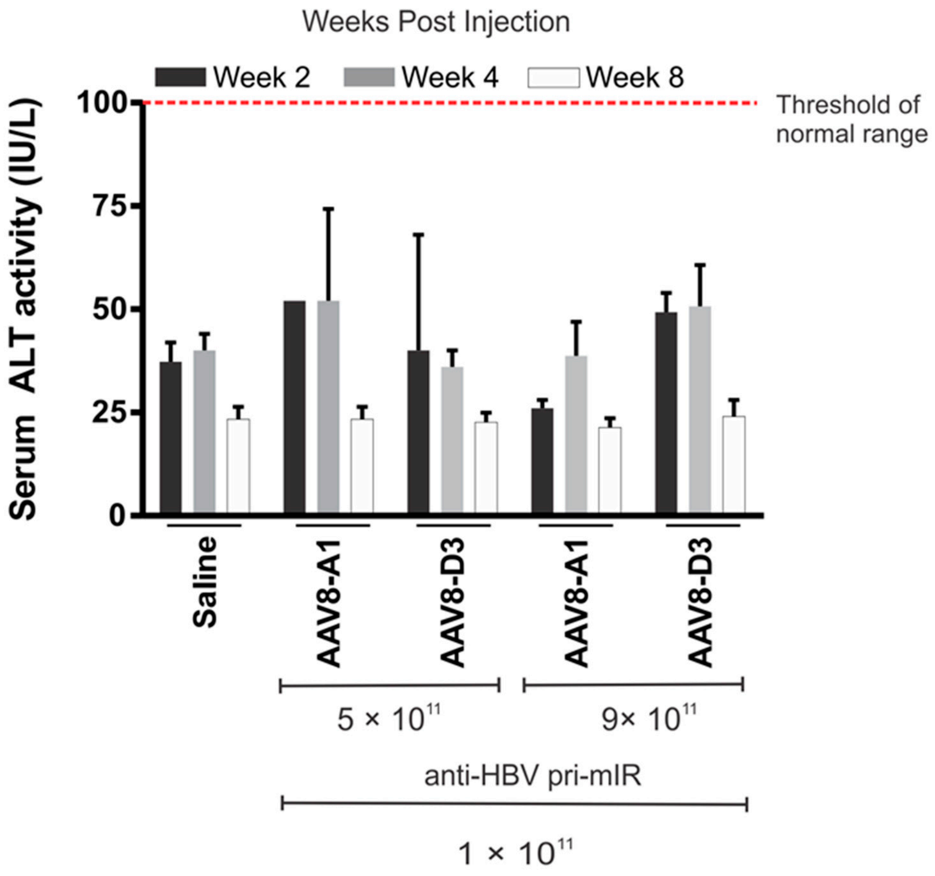

Figure S4. ALT activity in serum collected from mice treated with saline or co-infected with AAV8-HBV and anti-HBV AAV. Means and SEM were calculated from data obtained from four mice. The red dotted line indicates threshold of accepted normal range < 100IU/L.

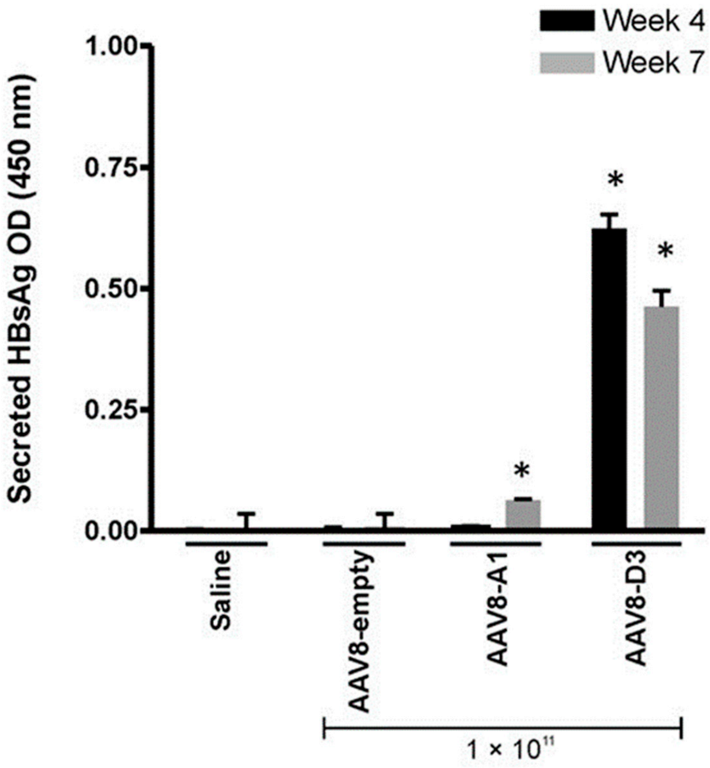

Figure S5: HBsAg expression in mice transduced with 1 × 10^{11} AAV8-HBV VPEs/mouse. Means and SEM were calculated from data obtained from 8 mice per group. Mice were bled and 100 µL undiluted serum used for HBsAg ELISA. Statistically significant differences between samples were calculated using Student's two tailed paired test. * $P \leq 0.05$ was considered statistically significant.
